# Reply to Stanojcic et al. Comment on “Fernández-Vigo et al. Objective Classification of Glistening in Implanted Intraocular Lenses Using Optical Coherence Tomography: Proposal for a New Classification and Grading System. *J. Clin. Med.* 2023, *12*, 2351”

**DOI:** 10.3390/jcm12113686

**Published:** 2023-05-26

**Authors:** José Ignacio Fernández-Vigo, Bárbara Burgos-Blasco, Lucía De-Pablo-Gómez-de-Liaño, Inés Sánchez-Guillén, Virginia Albitre-Barca, Susana Fernández-Aragón, José Ángel Fernández-Vigo, Ana Macarro-Merino

**Affiliations:** 1 Centro Internacional de Oftalmología Avanzada, 28010 Madrid, Spain; depablo.lucia@gmail.com (L.D.-P.-G.-d.-L.); susanaferar@gmail.com (S.F.-A.); jose.fernandez-vigo@fernandez-vigo.com (J.Á.F.-V.); ana.macarro@fernandez-vigo.com (A.M.-M.); 2 Department of Ophthalmology, Hospital Clínico San Carlos, Instituto de Investigación Sanitaria (IdISSC), 28040 Madrid, Spain; bburgos171@hotmail.com; 3 Department of Ophthalmology, Hospital Universitario 12 de Octubre, 28041 Madrid, Spain; 4 Department of Ophthalmology, Hospital Perpetuo Socorro, 06010 Badajoz, Spain; inessanchezguillen@gmail.com; 5 Centro Internacional de Oftalmología Avanzada, 06011 Badajoz, Spain; 6 Department of Ophthalmology, Universidad de Extremadura, 06006 Badajoz, Spain

We appreciate the comments made by Stanojcic et al. [1] regarding our recently published article in which we report the assessment of the glistening in intraocular lenses (IOL) using swept-source optical coherence tomography (SS-OCT) [2].

We agree that slit-lamp (SL) photography has been the gold standard technique for glistening evaluation to date, although it has several drawbacks [3,4,5]. It requires complex post-processing of the images, which is a time-consuming method and requires an expert evaluator. Therefore, it is not a quick exploration and quantification method and cannot be carried out during the clinical visit. Moreover, SL photography has greater variability depending on the light conditions (flash or background light), plane, angle and size of the slit, pupillary dilation and media transparency. Hence, areas that are slightly out of focus are not visualized accurately and the lighting on the different planes due to the thickness of the lens will not be totally homogeneous, so some glistening may go unnoticed and will not be accounted for. Therefore, even if performed in a standardized manner, there is enormous variability depending on the criteria of the person taking the photo and the examiner who evaluates and quantifies the glistening.

Conversely, our newly proposed method using SS-OCT is simple, fast, objective and reproducible, allowing us to directly quantify the presence and severity of the glistening in vivo [2]. Even when the repeated images in our study were acquired without any eye-tracking and were performed manually, the repeatability of the quantification and classification of the glistening on the IOL was excellent. However, as with all exploration methods, OCT has some limitations. We agree with Stanojcic et al. that the assessment of the smaller microvacuoles (MVs) using OCT is limited by the device’s resolution, as we previously recognized. An underestimation of the total number of MVs could thus be obtained, especially those smaller than 8 µm. Nevertheless, a study reported that only MVs greater than 10 µm induce a worsening in the modulation-transfer function [6], and Henriksen et al. concluded that the glistening area, at a key size, was correlated with random light scatter [7]. In addition, as Philippaki et al. reported in their study, the median size of the MVs in their laboratory study analyzing the same IOL as in the present study was 23.8 µm, which was easily identifiable using OCT [8].

In this original study, in which we proposed the OCT method for glistening assessment, we only analyzed one central pupillary scan, but we observed an excellent correlation in the quantification of the IOL glistening between the horizontal and vertical scans (R ≥ 0.834; *p* < 0.001). Interestingly, one additional advantage of OCT is that it is possible to perform a raster or cube exam to analyze almost all the central area of the lens after pupil dilation. Therefore, the quantification and distribution study, for example, using heat maps, should be assessed in future studies. It should be noted that most authors that quantify IOL glistening using SL-photography only quantify a small area (1 mm^2^).

Regarding the details of the exploration and quantification using SS-OCT, the examination was performed in a room under standardized mesopic lighting conditions set at 7 EV or 320 lux using a lightmeter (Flash mate K-308S, SEKONIC), the luminance of the screen was 600 nits, and the resolution was 1920 × 1080 pixels. As we have also previously stated, we counted the MVs without any modification on the original OCT image. In a pilot study, we checked that by changing the luminance of the image, the MV counting did not differ significantly. We did observe, however, that the signal strength in which the OCT scan was captured correlated with the number of MVs detected.

As for the possible artefacts on the images, it was previously stated that images with poor quality were excluded from the study. The high-reflectivity structure on the IOL surface in Figure 1B of our original published paper that Stanojcic commented on is the capsulorhexis, which is not in the visual axis which is the relevant area to be studied. We agree that the capsulorhexis may impair the detection of underlying MVs as well as corneal opacities or the lack of pupil dilation, but these factors will affect both SS-OCT and SL photography.

Future studies in which a direct comparison of the quantification and classification systems between SL photography (based on a frontal plane of the IOL) and OCT (based on a transversal plane of the IOL) would be very interesting, but it is not easy to assess the same area precisely. In this regard, the en face OCT modality, if available in the future for the anterior segment, could be a great tool for performing a direct comparison on the same IOL plane [9].

Finally, we agree that manual quantification of the glistening is a time-consuming method that should be surpassed by automatic counting. Therefore, we are working on the development of a deep-learning-based algorithm that could assess the number, size, shape, distribution, and reflectivity of the IOL glistening, and we hope that this will result in a great advance for the assessment and follow-up of IOL glistening in clinical practice.

## Data Availability

Data used to support the findings presented in this study are available on request from the corresponding author.
